# Age-Related Changes of Intraocular Pressure in Elderly People in Southern China: Lingtou Eye Cohort Study

**DOI:** 10.1371/journal.pone.0151766

**Published:** 2016-03-17

**Authors:** Xiaotong Han, Yong Niu, Xinxing Guo, Yin Hu, William Yan, Mingguang He

**Affiliations:** 1 State Key Laboratory of Ophthalmology, Zhongshan Ophthalmic Center, Sun Yat-sen University, Guangzhou, China; 2 Guangzhou No.11 People’s Hospital, Guangzhou, China; 3 Centre for Eye Research Australia, University of Melbourne, Melbourne, Australia; National Eye Institute, UNITED STATES

## Abstract

**Purpose:**

To study age-related changes of intraocular pressure (IOP) and assess the cohort effect in both cross-sectional and longitudinal settings among elderly Chinese adults.

**Methods:**

Participants were enrolled from the Lingtou Eye Cohort Study with Chinese government officials aged 40 years and older at baseline and received physical check-up and ocular examinations from 2010 to 2012. IOP was measured using a non-contact tonometer according to standardized protocols, as well as systolic blood pressure (SBP), diastolic blood pressure (DBP) and body mass index (BMI). Participants who had attended IOP measurements in both 2010 and 2012 were included in this study. Cross-sectional association of IOP with age was assessed using multivariate liner regression analyses and based on the data of 2010. Longitudinal changes in IOP were assessed by paired t-test.

**Results:**

A total of 3372 subjects were enrolled in the current analysis (2010 mean [SD] age, 61.9 [7.1] years; 60.2% men). The mean IOP in 2010 was 15.4±2.3 mmHg for women and 15.2±2.3 mmHg for men with an intersex difference (P = 0.029). Cross-sectional analysis showed that IOP was negatively associated with age (P = 0.003, β = -0.033 for women and P<0.001, β = -0.061 for men) adjusted for baseline SBP, DBP and BMI. Paired t-test suggested that IOP was higher in the year 2012 than 2010 in women (P = 0.006) but did not change significantly in men within 2 years (P = 0.345). In addition, the 2-year changes of IOP were not associated with age adjusted for baseline IOP in 2010 (P = 0.249).

**Conclusion:**

Cross-sectional data suggests that IOP is lower in people with older age. Longitudinal data does not support such findings and thus the identified decreasing pattern with age in cross-sectional analysis is likely caused by cohort effects.

## Introduction

Elevated intraocular pressure (IOP) is a major, and currently the only modifiable risk factor for glaucoma, a common disease and leading cause of irreversible blindness worldwide.[1] Age has also been established as a significant contributing factor to glaucoma.[2] The relationship between IOP and age has been previously investigated in many cross-sectional studies. Studies based on European or American populations mostly reported an increase of IOP with age, such as in the Beaver Dam Eye Study and the Barbados Eye Study. [3, 4] On the other hand, a decreasing trend of IOP with age in Asian people has been reported in a majority of studies. The Shihpai Eye Study in Taiwan, the Tajimi Eye Study in Japan and the Healthy Twin and the GENDISCAN Study of Korean and Mongolian populations all reported a negative association between IOP and age.[5–7] This discrepancy was explained as secondary to ethnic and environmental influences.[6]

Cross-sectional studies are susceptible to cohort effects when investigating for age effects; that is, an essential selection bias exists in different birth cohorts of the study population due to different environmental and social exposures. Therefore longitudinal studies may present an advantage in illustrating any true underlying associations. However, longitudinal studies of IOP change are rare and show varying results.[8–12] Further research and data, especially from longitudinal studies, are needed to assess the relationship between changes in IOP and age.

A variety of factors have previously been proposed and demonstrated to be associated with IOP. Body mass index (BMI) and systolic blood pressure (SBP) were the most frequently reported factors from previous studies all over the world.[13–16] These should be taken into consideration when investigating the relationship between IOP and age as they are potential confounders. In this paper, we aimed to investigate age-related changes of IOP in both cross-sectional and longitudinal settings and to identify the impact of cohort effect on current cross-sectional analysis.

## Materials and Methods

### Study population

The study participants were enrolled from the Lingtou Eye Cohort Study, which has been described in detail elsewhere.[17] In brief, government employees aged 40 years and older without history of major cardiovascular events were recruited through the Guangzhou Government Servant Physical Check-up Center in 2008 for long-term follow-up study on account of their high retention rates for annual check-up. The study was conducted under the guidelines of the Declaration of Helsinki and approved by the Ethics Committee of the Zhongshan Ophthalmic Center, Sun Yat-Sen University, Guangzhou. Written informed consent was obtained from all participants.

The study was initiated in 2008 and included physical and ophthalmologic examinations, as well as questionnaire administered by face-to-face interview. Height, weight, SBP and diastolic blood pressure (DBP) were measured according to standardized protocols by trained nurses and detailed medical histories including ocular, systemic and surgical history (confirmed by medical records) were collected. All participants of the baseline survey were invited to attend the annual follow-up examinations. Follow-up examinations were the same as baseline and performed according to the standardized protocols.

Our study is an exploratory perspective study and included 3770 participants from the Lingtou Eye cohort study who had attended IOP measurement in both 2010 and 2012. Cross-sectional analysis was based on the IOP data initially measured in 2010 and longitudinal analysis was based on the data in 2010 and 2012 of six birth cohorts ranging from the 1930s to 1960s. We further excluded 150 (4.0%) who received IOP lowering treatment or had undergone corneal or intraocular surgery in at least one eye, and 241 (6.4%) whose IOP values were out of the normal range (10-21mmHg). Subjects in the birth cohorts of the 1920s and the 1970s were excluded due to a small sample size of 7 (0.2%) participants. Thus, 3372 (89.4%, 1342 women and 2030 men) were available for the present analysis.

### Measurement of intraocular pressure

IOP was measured in both eyes by non-contact tonometer (CT-80A computerized tonometer, Topcon Ltd., Japan) prior to pupil dilation. The data was saved as the mean of 3 continuous measurements. If the 3 consecutive measurements could not achieve SE < 5% or if the subject could not cooperate, the IOP was considered unreliable and further tested for two more times by a trained nurse to get a reliable value. If a reliable value couldn’t be reached at retest, the IOP value was then deemed as missing and not included in the analysis. One final reading was recorded for each eye.

### Measurement of blood pressure and body mass index

Blood pressure was measured following protocol using an automatic upper arm blood pressure monitor by trained nurses. Height and weight were measured with subjects wearing light clothes without shoes in the standing position using an automatic height and weight tester. Height was measured to the closest 0.5 cm and weight was measured to the closest 0.5 kg. BMI was calculated as weight in kilograms divided by height in meters squared.

### Statistical analysis

All data analysis was performed using Stata Package (Stata 8.0; Stata Corp, College Station, Texas, USA). Measurements from the right eye were selected for analysis because of the high correlation between the two eyes and were summarized using means and standard deviations (SD). Univariate and Multivariate liner regression models were used to investigate the cross-sectional associations between IOP and age. BMI, SBP and DBP were also included in the regression model as confounding factors. In the longitudinal analysis, participants were divided into six age groups by five-year intervals ranging from 50–54 years to 75–79 years. Paired t-test was used to compare the intraocular pressure of the same subject between 2010 and 2012 and a P-value of equal or less than 0.05 was considered statistically significant.

## Results

**Fig 1:**
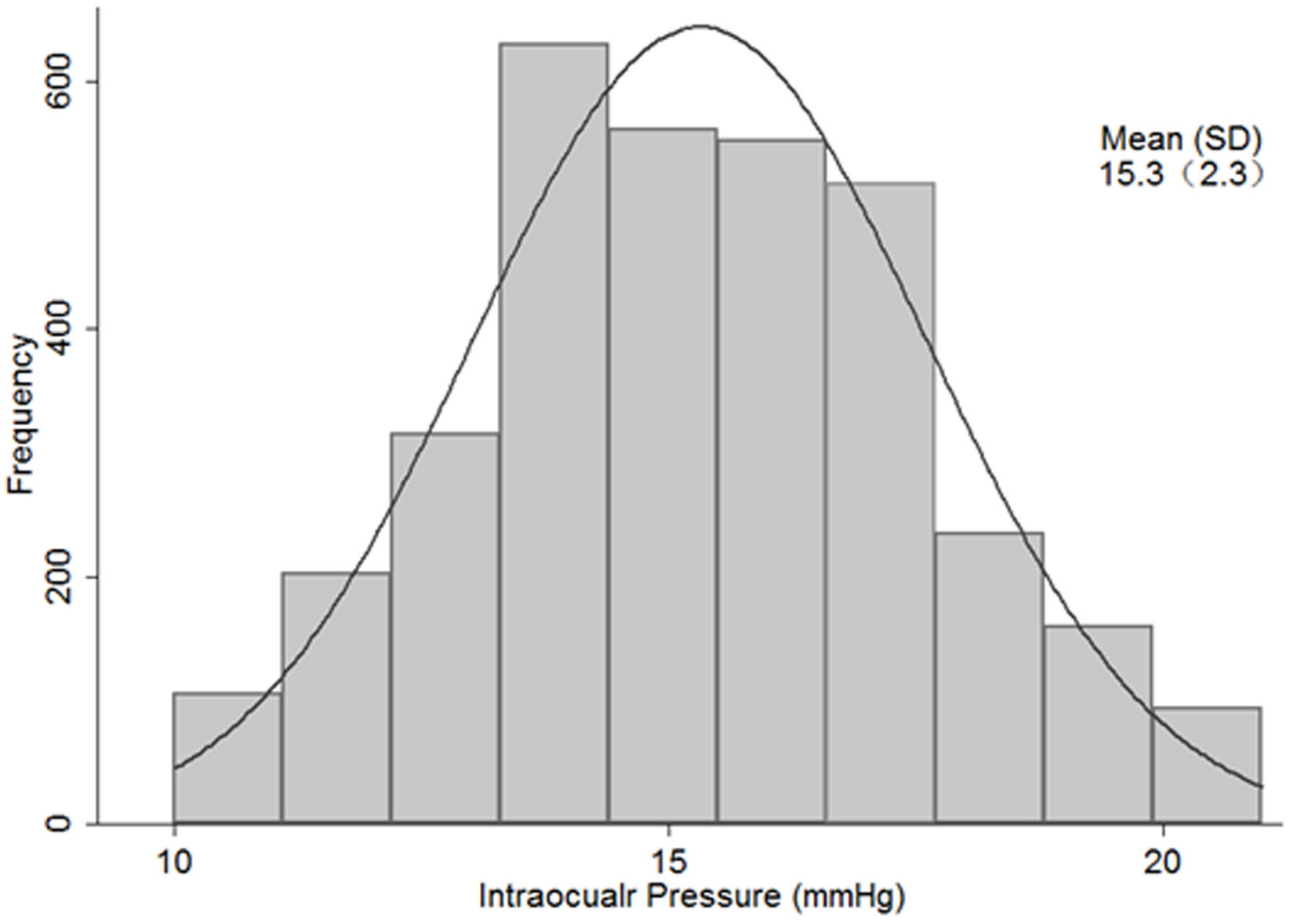
Distribution of intraocular pressure among Chinese adults in Lingtou, China (2010). Histogram of Intraocular pressure for the population under study at baseline. Right eye data was used and the total number is 3372. The dark grey curve represents the normal distribution.

Data of 3372 people were used for analysis of which 60.2% were men. Table 1 summarizes the baseline characteristics of the participants by birth cohorts. The mean IOP was 15.4±2.3 mmHg for women and 15.2±2.3 mmHg for men. Student’s t-test showed that women had higher mean IOP values than men in general (P = 0.029). Fig 1 illustrates the distribution of IOP for the population. IOP was nearly normally distributed with a peak at 14 to 15 mmHg.

**Table 1:** Baseline Characteristics of the Participants (mean ± standard deviation).

Birth Cohorts (years)	No. Of Subjects		Mean ± SD							
			IOP (mmHg)		SBP (mmHg)		DBP (mmHg)		BMI (kg/m^2^)	
	Male	Female	Male	Female	Male	Female	Male	Female	Male	Female
50–54	358	270	15.7±2.4	15.6±2.3	125.7±15.0	117.5±15.0	76.8±10.5	69.3±10.1	25.1±3.0	23.8±2.9
55–59	476	462	15.6±2.2	15.4±2.3	127.6±15.9	122.2±15.8	76.1±10.3	69.6±10.6	25.1±2.8	23.9±3.2
60–64	434	290	15.2±2.2	15.4±2.3	132.3±15.1	126.3±16.4	75.9±9.5	70.1±10.3	24.8±2.7	24.4±3.3
65–69	327	152	14.9±2.3	15.5±2.4	135.2±14.7	131.0±15.4	74.2±9.3	69.1±9.5	24.9±3.0	24.1±3.6
70–74	294	118	14.9±2.2	14.8±2.4	137.0±16.3	136.9±18.0	72.1±9.7	68.0±10.7	24.0±3.2	23.7±2.9
75–79	141	50	14.6±2.2	15.5±2.3	136.6±15.4	143.5±17.9	69.1±10.2	71.2±8.3	24.2±3.3	24.7±3.8
Total	2030	1342	15.2±2.3	15.4±2.3*	131.5±16.0	125.3±17.3**	74.8±10.2	69.5±10.3**	24.8±3.0	24.0±3.2**

IOP: intraocular pressure; SBP: systolic blood pressure; DBP: diastolic blood pressure; BMI: blood mass index.

P values for difference between males and females using group t-test:

* p<0.05;

** p<0.001

**Fig 2:**
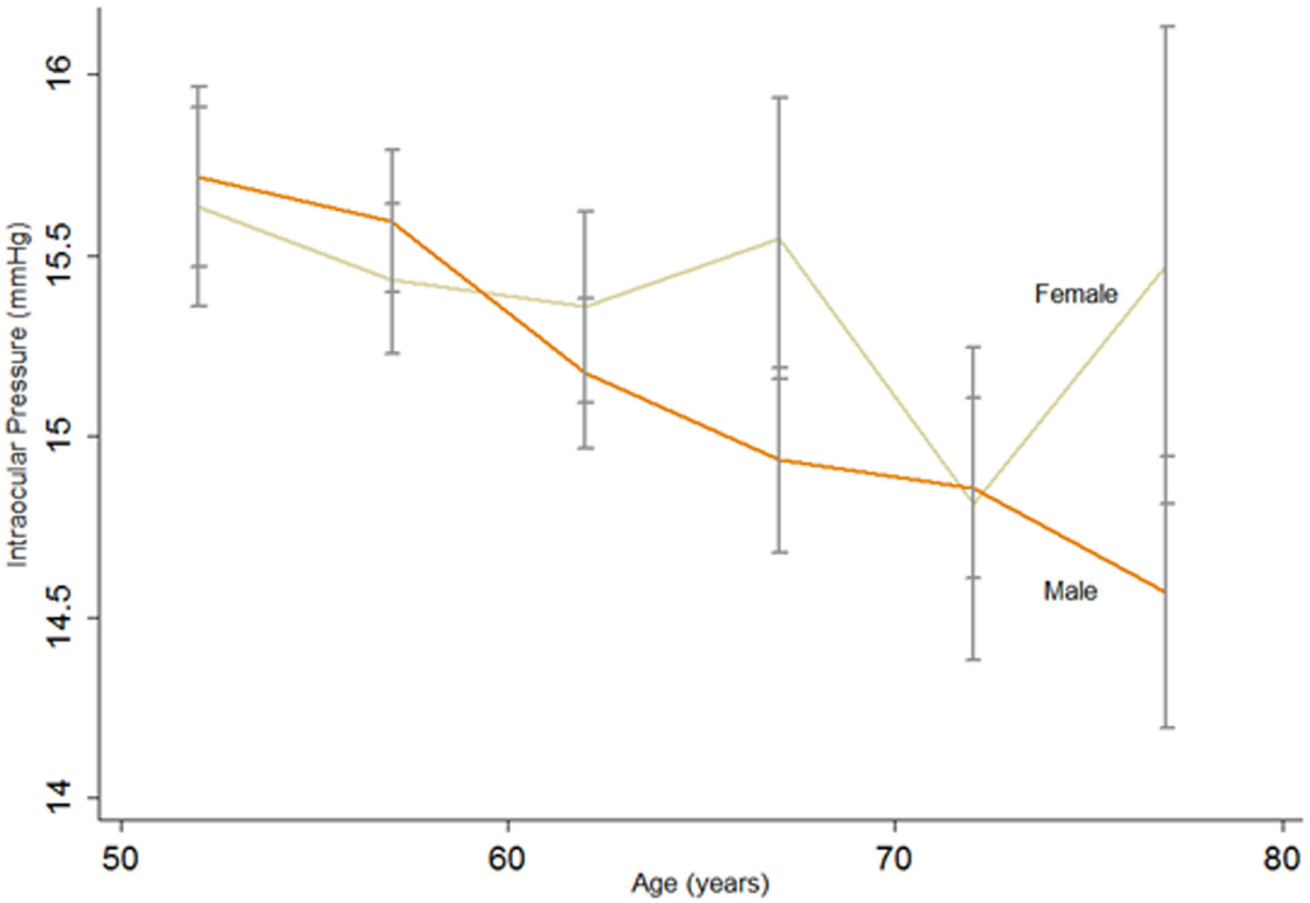
Effect of aging on intraocular pressure (IOP) in cross-sectional analysis. Cross-sectional change of IOP with increasing age for both genders. Each line was simply connected by 6 points which were the mean IOP value of the right eye of the six birth cohorts (50–54;55–59;60–64;65–69;70–74;75–79) from left to right, respectively. The error bars for each point show the upper and lower 95% confidence limits.

Cross-sectional analysis showed that IOP decreased significantly with age in men of all birth cohorts and women with the exception of the 65–69 birth cohort (Fig 2). Table 2 showed the cross-sectional associations of related risk factors of the year 2010 with IOP. Multiple liner regression showed IOP was negatively related with age after adjusting for SBP, DBP and BMI (P = 0.003, β = -0.033 for women and P<0.001, β = -0.061 for men).

**Table 2:** Cross-sectional Associations of Related Risk Factors with IOP in 2010 according to Univariate and multivariate Regression Analyses.

Factors	Univariate regression				Multivariate regression			
	Regression coefficients		P Value		Regression coefficients		P Value	
	Male	Female	Male	Female	Male	Female	Male	Female
Age(yrs)	-0.050(-0.063~-0.037)	-0.022(-0.041~-0.004)	<0.001	0.019	-0.061(-0.077~-0.046)	-0.033(-0.055~-0.109)	<0.001	0.003
SBP(mmHg)	0.016(0.010~0.022)	0.016(0.009~0.023)	<0.001	<0.001	0.022(0.013~0.031)	0.013(0.002~0.024)	<0.001	0.025
DBP(mmHg)	0.032(0.022~0.042)	0.033(0.021~0.045)	<0.001	<0.001	0.002(-0.012~0.015)	0.016(-0.000~0.033)	0.805	0.061
BMI(kg/m^2^)	0.075(0.041~0.110)	0.053(0.014~0.093)	<0.001	0.008	0.030(-0.005~0.065)	0.023(-0.018~0.064)	0.096	0.279

IOP: intraocular pressure; SBP: systolic blood pressure; DBP: diastolic blood pressure; BMI: blood mass index.

**Fig 3:**
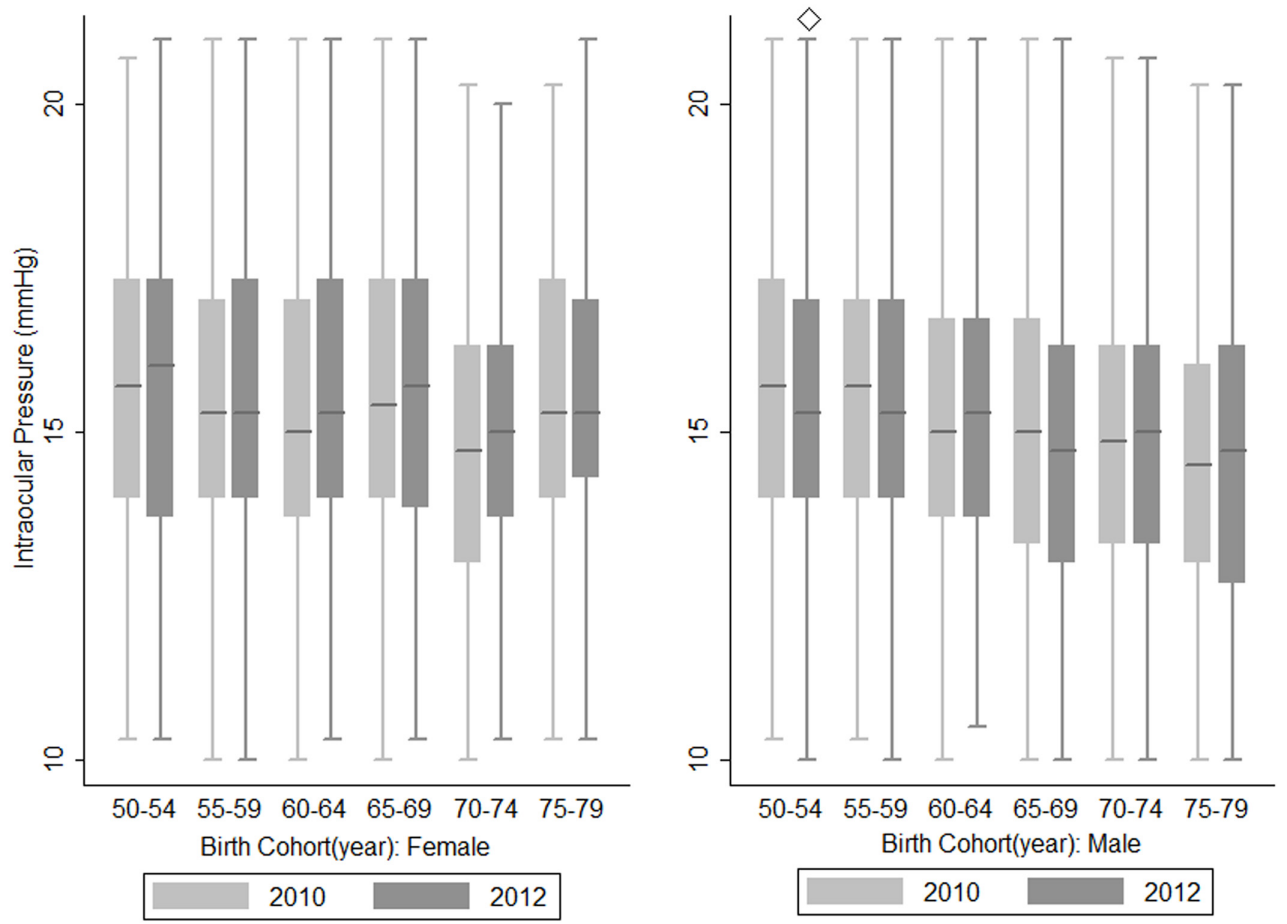
Comparison of intraocular pressure by sex and birth cohorts in 2010 and 2012. Box plots showing the IOP of the right eye between the year 2010 and 2012 in each birth cohort of the population, ◇ represents a P value <0.05 calculated by paired t-test comparing the mean IOP of the year 2010 and 2012.

Fig 3 shows the 2-year longitudinal changes of IOP by sex specific birth cohorts. Paired t-test showed there were no statistically significant differences in IOP of the study population between 2010 and 2012 of all birth cohorts, except for the 50–54 birth cohort in men which showed a lower IOP in 2012 (P = 0.006). Regarding sex, IOP increased significantly with age in women (P = 0.006) but remained relatively steady in men (P = 0.345). Furthermore, the 2-year changes of IOP were not associated with age after adjusting for baseline IOP (P = 0.249).

## Discussion

Our study is the first study to combine both cross-sectional and longitudinal analysis in a large Southern Chinese adult population, suggesting for the first time that the decreasing trend of IOP with age reported by most established cross-sectional studies in Asia is likely caused by cohort effects.

The mean IOP reported in our study was similar to the Liwan Eye Study which was also based on a Southern Chinese adult population (15.4±3.1 mmHg for women; 15.0±3.2 mmHg for men), and the Beijing Eye Study which was based on a Northern Chinese elderly population (15.6±3.0 mmHg),[18] but higher than the reported mean IOP in Japanese studies. Fukuoka et al. reported a mean IOP of 14.1±2.3 mmHg based on the Tajimi Eye Study while Nomura reported a lower IOP value in a larger Japanese study population (11.5±2.4 mmHg for women; 11.9±2.5 mmHg for men).[6, 9] Regarding sex, most studies reported that women had a higher IOP than men,[5, 8] while others found no difference between the sexes.[6] Our study reported a higher IOP in women, and although the underlying mechanisms are unknown, one possible explanation relates to the changing aqueous production with hormonal differences and the onset of menopause.[19]

Most American and European studies, both cross-sectional and longitudinal in nature, conclude that IOP increases with age. One study suggested that age-related structural changes in the trabecular meshwork substantially counteracts the reduced production of aqueous humor with age.[8, 15, 20] While there have been fewer studies based on African populations, a study in West Cameroon and the well-known Barbados Eye study also reported this association.[21, 22] The majority of studies in Asia however, have reported the opposite trend.[5, 7, 18] The Handan Eye Study in China reported a reversed U like course interaction between IOP and age.[13] This was discussed in other studies as being due to a “survival effect”. A smaller proportion of obese and hypertensive subjects exist in the elderly groups due to the increased mortality of cardiovascular disease, and therefore IOP would seem to be lower with age in the absence of these risk factors, forming the rear decline of this curve.[23]

The inconsistency of Asian study findings with other, similar studies was noted by Yoshida and Fukuoka and attributed to ethnic and environmental effects. They proposed that the hypertensive effects caused by high BP and BMI in Europeans and Americans outweighed the hypotensive effects caused by age, causing IOP to seemingly increase with age. Conversely, as the prevalence of obesity and hypertension is lower in Japan, the hypotensive effects of age might predominate, resulting in an apparent decrease in IOP with age. [6, 23] Our cross-sectional analysis supports this and validates the negative correlation of IOP with age reported by most Asia studies. This finding persisted after adjusting for both blood pressure and BMI.

Few longitudinal studies of IOP and age exist in the literature, and even fewer of Asian populations, and no consensus has been reached at present. Nomura reported a significant increase in IOP with age in a 9-year longitudinal follow-up, while Nakano reported a decreasing trend of IOP with age in all age groups over a 10-year follow-up.[9, 11]

In our longitudinal analysis, IOP was found to increase with age in women over the two-year follow up between 2010 and 2012, but not found to change in men, which is inconsistent with the cross-sectional results. This inconsistency was supported by another Japanese cross-sectional and longitudinal study.[9] It is challenging for cross-sectional studies to separate the effects of developmental influences from cohort effects when examining across a wide range of ages. Cohort effects may influence population data, as people who are born at similar times are exposed to intrinsically similar events and demographic trends in life, making the study group unique and different from other population groups. In particular, this manifests as different lifestyle and environmental factors affecting educational levels, nutritional intake and exercise habit.

To the best of our knowledge, this is the first longitudinal study to report on the age-related changes of IOP in Southern China and the strengths include the relatively large population size. Nevertheless, some limitations of this study should be taken into consideration. The Goldmann Applanation Tonometer (GAT), which is widely believed to be the gold-standard measurement tool, was not used in this study as NCT does not require corneal contact or anesthesia and is more efficient at measuring large populations. Though reported to be reliable for values within the normal IOP range, the test-retest repeatability was ± 2.8 mmHg for CT80 non-contact tonometers from previous studies and may affect the validity of IOP results compared to other studies. [5],[24–26] Central corneal thickness (CCT) has a significant association with IOP but unfortunately was not measured in our study due to restrictions of the hospital.[27, 28] Lastly, as the IOP measurements in our study were not performed at the same time of day for different visits due to logistical difficulty, the comparability of recordings may be affected by diurnal fluctuations. The circadian fluctuations of IOP can be substantial in healthy elderly adults and also independent of mean CCT or CCT fluctuations in glaucoma patients.[29, 30] However the largest fluctuation mostly occurred during the night whereas all the examinations in our study was taken place at office hours (8:30–12:00 am and 2:30–5:00 pm) and the majority of participants came to the physical check-up center at a routine time, thus enhances comparability.[30, 31]

As this study is of a community cohort with the majority of subjects having IOPs within a normal range, the study conclusions may not be directly inferable to the general population. Besides, the two-year observational time is relatively short and we can not draw a definite conclusion of the age related changes of IOP. We are following this group of participants and further findings from longitudinal observations will be reported. However, the combined cross-sectional and longitudinal studies on this large population cohort offers substantial evidence and an important illustration of the size of cohort effects.

In summary, the distribution of IOP in our study was found to be similar to that of most studies based in East Asian populations. The discrepancy between cross-sectional and longitudinal analysis suggests the decreasing trend of IOP with age reported in many studies is likely due to cohort effects. Cohort effects should be taken into consideration while investigating time-related variables such as age. Future population-based studies with prolonged follow-up would aim to illustrate the relationship between IOP and age to best address the resource demands of glaucoma in an aging society.

## Supporting Information

S1 Dataset This is the dataset for this article. (XLS)
